# Risk Factors of Ankle Sprain in Soccer Players: A Systematic Review and Meta-Analysis

**DOI:** 10.3390/sports13040105

**Published:** 2025-03-28

**Authors:** Amir Human Hoveidaei, Amir Reza Moradi, Amin Nakhostin-Ansari, Mohammad Mehdi Mousavi Nasab, Seyed Pouya Taghavi, Shayan Eghdami, Bijan Forogh, Masumeh Bagherzadeh Cham, Christopher J. Murdock

**Affiliations:** 1 International Center for Limb Lengthening, Rubin Institute for Advanced Orthopedics, Sinai Hospital of Baltimore, Baltimore, MD 21215, USA; hoveidaei.a.h@gmail.com; 2 School of Medicine, Tehran University of Medical Sciences, Tehran 1416634793, Iran; moradi-ar@student.tums.ac.ir; 3 Neuromusculoskeletal Research Center, Department of Physical Medicine and Rehabilitation, School of Medicine, Iran University of Medical Sciences, Tehran 1416634793, Iran; a-nansari@alumnus.tums.ac.ir (A.N.-A.); eghdami.s@iums.ac.ir (S.E.); bagherzadehcham.m@iums.ac.ir (M.B.C.); 4 School of Medicine, Shahid Beheshti University of Medical Sciences, Tehran 1983969411, Iran; mehdi1379mmmn@gmail.com; 5 Student Research Committee, Kashan University of Medical Sciences, Kashan 8715981151, Iran; taghavi-p@kaums.ac.ir; 6 Department of Orthopaedic Surgery, Johns Hopkins University, Baltimore, MD 21205, USA; cmurdoc5@jhmi.edu

**Keywords:** ankle sprain, football, risk factors, soccer

## Abstract

Background: Soccer is associated with substantial injury risk, with reported between 13 to 35 injuries per 1000 player-hours of competitive play. Notably, approximately 77% of soccer-related ankle injuries are attributed to ankle sprain injuries (ASIs). ASI can lead to chronic ankle instability, obesity, and post-traumatic osteoarthritis. This study focuses on identifying factors such as gender, age, body mass index (BMI), and a history of ASIs, which contribute to the development of ASI in soccer players. Methods: A systematic literature search was conducted in October 2023 across databases, including PubMed, Web of Science, Scopus, Cochrane Library, and ProQuest, without applying any filters. Keywords included ankle, ankle joint, sprain, risk factors, etc. Data extraction was performed on the included studies, with findings standardized and analyzed using Stata Statistical Software: Release 17 to determine a weighted treatment effect. Results: Our systematic review included 26 studies. The meta-analysis revealed that a history of ankle sprain is the most significant risk factor for future ASIs. BMI emerged as a risk factor in three out of seven studies, while age and height were significant in one out of six studies each. Gender and weight were not found to significantly affect ASI occurrence. Other factors identified but not subjected to a meta-analysis due to methodological heterogeneity or insufficient studies included playing surface, joint laxity, muscle weakness, match congestion, strength asymmetries, ground reaction forces, balance maintenance, skill level, and playing position. Conclusions: This research contributes valuable insights into the prevention of ASIs in soccer, highlighting the importance of previous ankle sprains and playing surface quality. These findings assist sports professionals in developing optimal conditions and strategies for effective ankle sprain prevention.

## 1. Introduction

Soccer is widely recognized for its high injury rates, with studies reporting 13 to 35 injuries per 1000 player-hours of competitive play [1]. Among these, ankle sprain injuries (ASIs) stand out as the most prevalent form of injury encountered by youth soccer players [2,3,4]. It is noted that a substantial 77% of ankle injuries within soccer can be attributed to ASI, highlighting the significance of this issue [5]. The investigation into the etiological factors that lead to foot and ankle injuries in soccer is not only crucial for understanding the nature of these injuries but also for developing preventive measures [6].

There is compelling evidence suggesting that individuals with a history of an ASI are at twice the risk of a subsequent ASI within at least one year of the initial occurrence, pointing to a cycle of vulnerability and re-injury [7,8]. Furthermore, chronic ankle instability, obesity, and post-traumatic osteoarthritis represent some of the long-term repercussions of ASI, although these are not exhaustive [7,9,10,11]. Interestingly, neuromuscular training (NMT) warm-up routines have been shown to significantly lower the incidence of acute lower extremity injuries in youth sports by 29 to 60 percent [12,13,14,15,16], indicating the potential for preventive strategies.

Soccer players face unique risk factors for ankle sprains due to the sport’s specific demands; yet, the literature lacks a focused synthesis of these factors. This systematic review and meta-analysis fills this gap, providing novel insights into both intrinsic and extrinsic risk factors to guide targeted prevention strategies and enhance player safety [17,18]. Identifying these factors is essential for formulating targeted ankle sprain prevention methods for young players.

This study aims to explore various potential risk factors for ASI, including gender, age, body mass index (BMI), and previous ASI history, among youth soccer players. By examining these elements in a systematic review, the research seeks to contribute valuable insights into the prevention and management of ASI in soccer.

## 2. Materials and Methods

**Systematic review protocol**

This systematic review was conducted in strict adherence to the Preferred Reporting Items for Systematic Reviews and Meta-Analyses (PRISMA) Protocols guideline [19], with its protocol registered under PROSPERO (CRD42022309294).

**Eligibility criteria**

Our study exclusively incorporated randomized controlled trials and prospective cohort studies that delve into the risk factors for ankle sprains among soccer players. We considered studies spanning various leagues, focusing on athletes who have encountered either a primary or subsequent ankle sprain injury in the context of official matches or training sessions. Eligible injuries included both contact and non-contact incidents leading to ligament stretching or tearing, contributing to ankle instability.

We excluded investigations related to other forms of ankle injuries, such as Achilles tendon ruptures, ankle fractures, peroneal tendonitis, tarsal tunnel syndrome, and osteochondral lesions, to maintain a focused inquiry into ankle sprains. The requirement for study inclusion was publication in English, with a clear disclosure of participant numbers, detailed injury data, follow-up criteria, and the statistical methods employed. Studies targeting non-soccer athletes, those centered on American football or rugby players, and research focusing on traumatic bony injuries, fractures, contusions, and muscle strains were deemed outside the scope of this review.

**Search strategy and outcome measures**

A comprehensive search through electronic databases, including PubMed, Web of Science, Scopus, Cochrane Library, and ProQuest, was conducted up to October 2023, following a predefined strategy. The search utilized the following keywords: “Ankle [Mesh]”, “Ankle Joint [Mesh]”, “Lateral Ligament, Ankle [Mesh]”, “Sprains and Strains [Mesh]”, “Ankle Injuries [Mesh]”, and “Risk Evaluation and Mitigation [Mesh]” (Appendix A). The search, executed by one author, utilized the Rayyan web tool for managing the identified records [20]. Our metrics for assessing the impact of risk factors on ASI were effect size, mean difference, and odds ratio.

**Study selection**

Titles and abstracts were initially screened by two authors for relevance, followed by the removal of duplicates and the retrieval of full-text articles for in-depth evaluation against our inclusion criteria. Discrepancies were resolved through discussion or consultation with a third author. Reference lists of selected articles were also reviewed to identify additional relevant studies.

**Data extraction and quality assessment**

Following study selection, two researchers independently conducted data extraction using a standardized form, capturing details such as study design, participant characteristics, interventions, risk factors, and outcomes. The Joanna Briggs Institute (JBI) critical appraisal tool was employed to assess the risk of bias across four domains: patient selection, index tests, reference standards, and flow and timing [21]. Quality assessment of RCT studies was conducted based on Cochrane tools. Any disagreements were resolved via discussion with a third author.

**Evidence synthesis**

To synthesize the evidence, we employed textual descriptions, tabulation, and data standardization using Stata software (StataCorp. 2021. Stata Statistical Software: Release 17. College Station, TX, USA: StataCorp LLC), ensuring a comprehensive analysis. Missing data were addressed using Wan’s statistical method, which considers sample size, median, and variability (range and interquartile range) to estimate missing values accurately [22].

Subgroup analysis was conducted to explore clinical heterogeneity and enhance the understanding of the data. The synthesis of the findings was presented as odds ratios (ORs) and risk ratios (RRs) with 95% confidence intervals (CIs) for dichotomous outcomes and mean difference (MD) for continuous outcomes. To ensure the reliability of our results, we used data from the longest follow-up period for each specific outcome in the included study code.

## 3. Results

**Figure 1:**
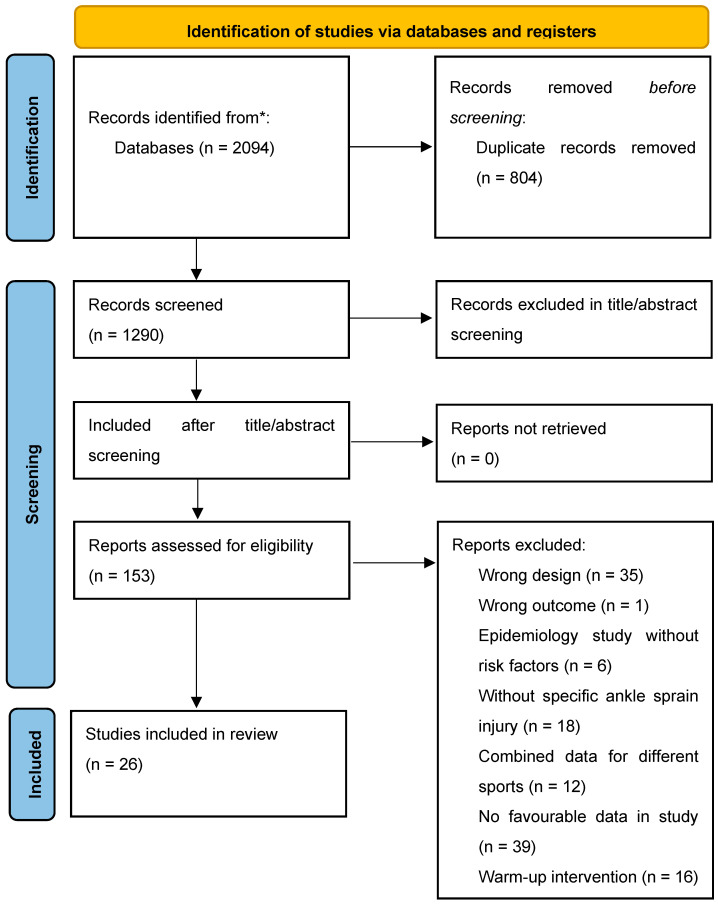
PRISMA flow diagram of risk factors of ankle sprain in soccer players.

Based on our search strategy, 1811 unique titles were obtained from four electronic databases. Further application of the inclusion and exclusion criteria narrowed the selection down to 26 studies for detailed data extraction (Figure 1). The details of each of the papers are shown in Table 1.

**Generalized joint hypermobility**

Among the risk factors analyzed, generalized joint hypermobility was investigated in two studies [31,41]. One study [41] found no significant difference in ankle sprain incidence related to joint hypermobility, while the other [31] suggested joint laxity could potentially increase the risk of ankle sprain (odds ratio [OR] = 3.38 [0.82–14.00]; *p* = 0.093), indicating a possible but uncertain risk factor.

**Age**

**Figure 2:**
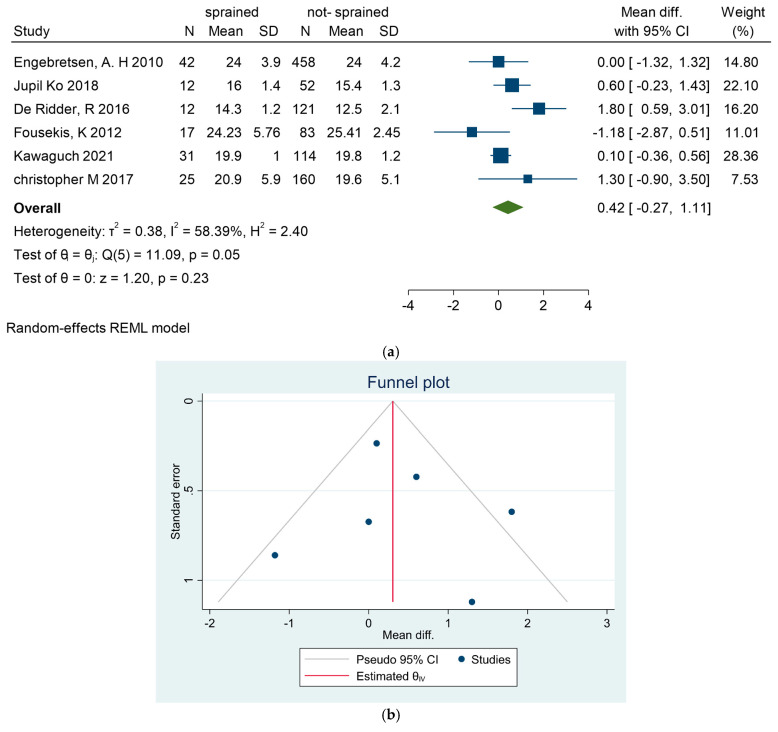

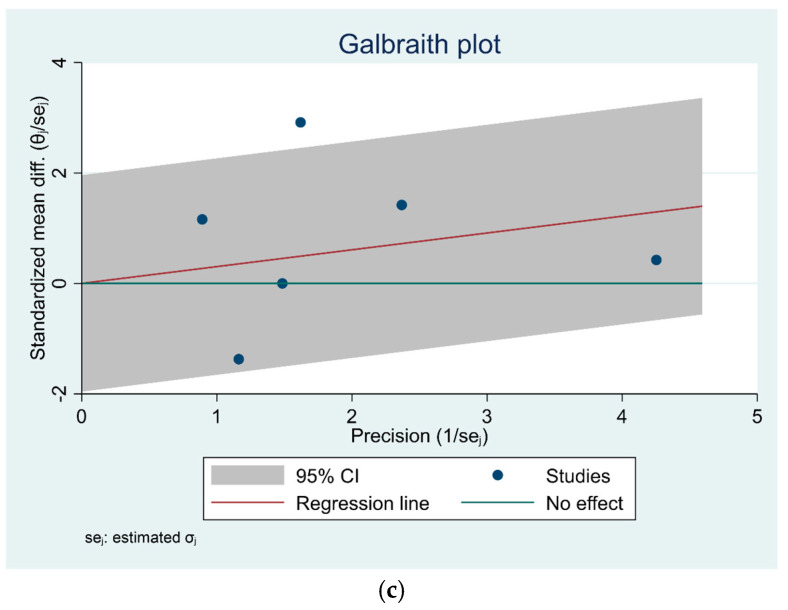
(**a**) Forest plot for age as a risk factor of ankle sprain in soccer players. (**b**) Funnel plot for age as a risk factor of ankle sprain in soccer players. (**c**) Galbraith plot for age as a risk factor of ankle sprain in soccer players [25,29,31,34,35,38].

Age was evaluated in six articles [25,29,31,34,35,38] as a potential risk factor for ASI. Only one study [25] identified age as a significant risk factor (*p* < 0.001). Overall, age did not significantly influence ASI risk (mean difference [MD] = 0.36; 95% CI = −0.27, 0.99), as illustrated in Figure 2a (mean difference), Figure 2b (funnel plot), and Figure 2c (Galbraith plot).

**BMI**

**Figure 3:**
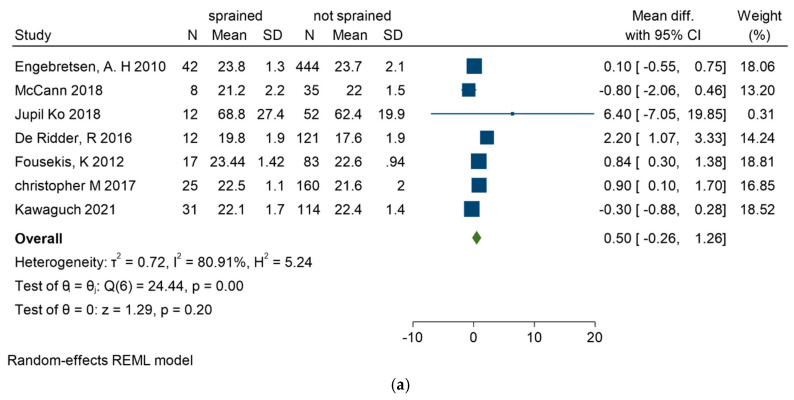

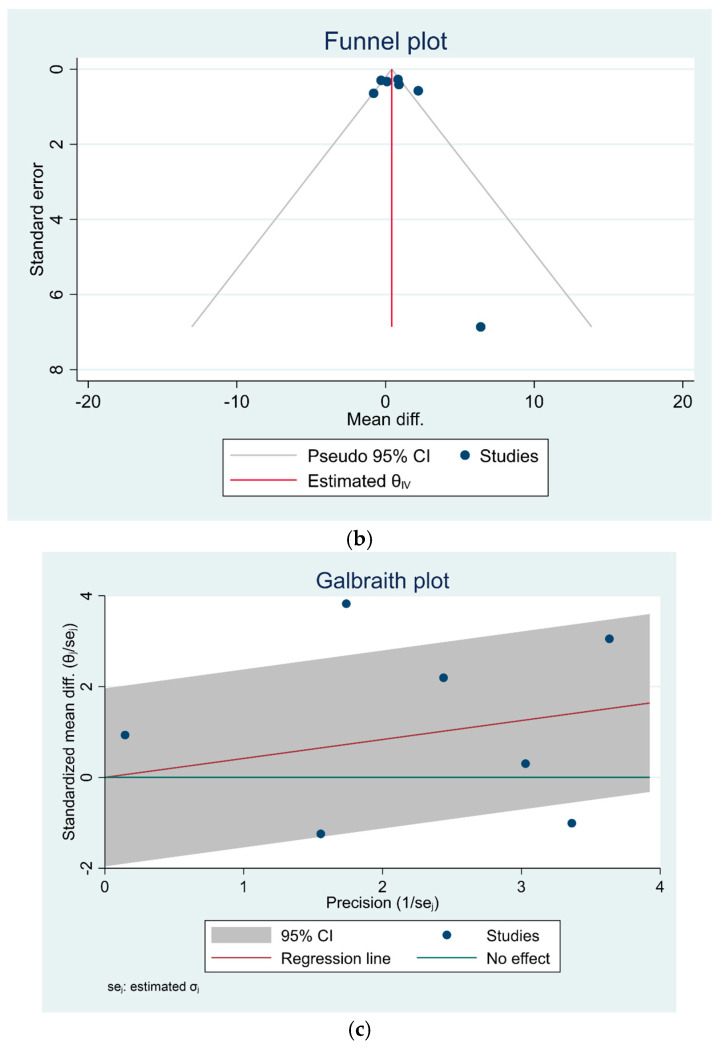
(**a**) Forest plot for BMI as a risk factor for ankle sprain in soccer players. (**b**) Funnel plot for BMI as a risk factor for ankle sprain in soccer players. (**c**) Galbraith plot for BMI as a risk factor for ankle sprain in soccer players [25,29,31,34,35,37,38].

Seven studies in total [25,29,31,34,35,37,38] examined BMI’s role, detailed in Figure 3a (forest plot), Figure 3b (funnel plot), and Figure 3c (Galbraith plot). Three studies [25,31,38] highlighted BMI as a risk factor for ASI. Specifically, De Ridder et al. [25] reported the most significant difference in BMI between injured and uninjured groups, with injured players having higher BMIs (19.8 ± 1.9 vs. 17.6 ± 1.9; *p* < 0.001).

**Weight**

**Figure 4:**
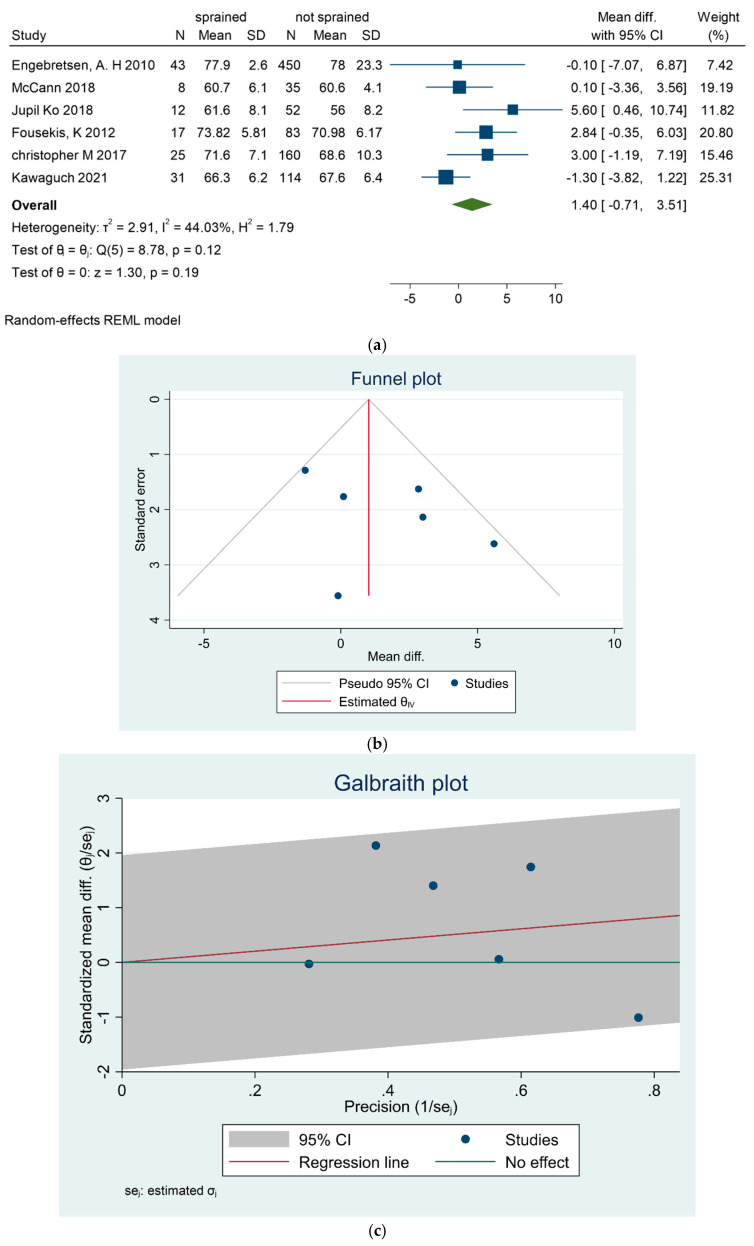
(**a**) Forest plot for weight as a risk factor of ankle sprain in soccer players. (**b**) Funnel plot for weight as a risk factor of ankle sprain in soccer players. (**c**) Galbraith plot for weight as a risk factor of ankle sprain in soccer players [25,29,31,34,37,38].

No significant correlation between player weight and ASI was found across studies [25,29,31,34,37,38]. The overall MD in weight between injured and uninjured groups was 1.4 kg [−0.71, 3.51], as shown in Figure 4a–c.

**Height**

**Figure 5:**
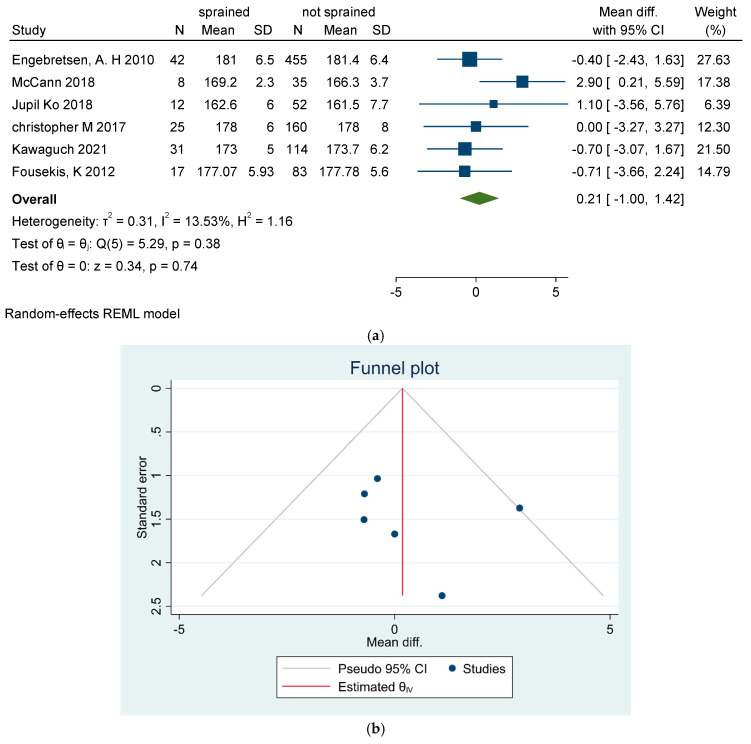

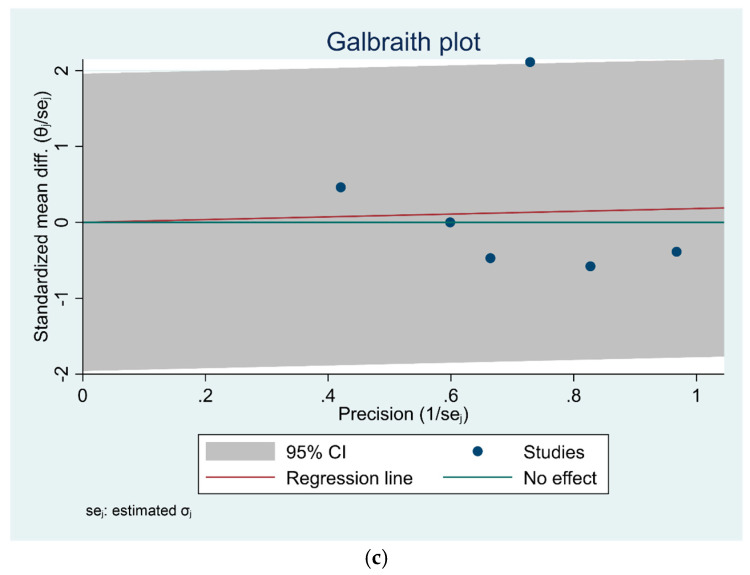
(**a**) Forest plot for height as a risk factor of ankle sprain in soccer players. (**b**) Funnel plot for height as a risk factor of ankle sprain in soccer players. (**c**) Galbraith plot for height as a risk factor of ankle sprain in soccer players [29,31,34,35,38].

Height was considered a risk factor in one study by McCann et al. [37], which found taller players to be more prone to ASI (*p* = 0.01). However, this finding was not corroborated by other studies [29,31,34,35,38], as depicted in Figure 5a–c.

**Sex**

**Figure 6:**
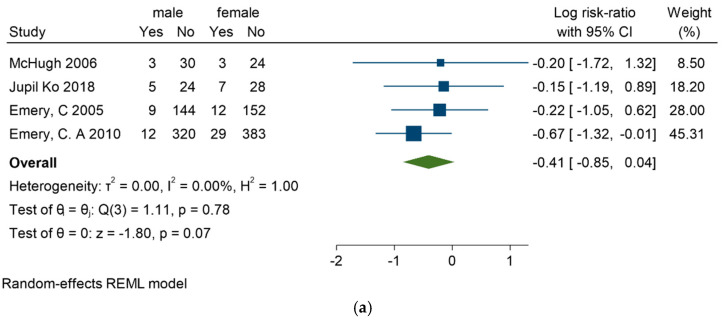

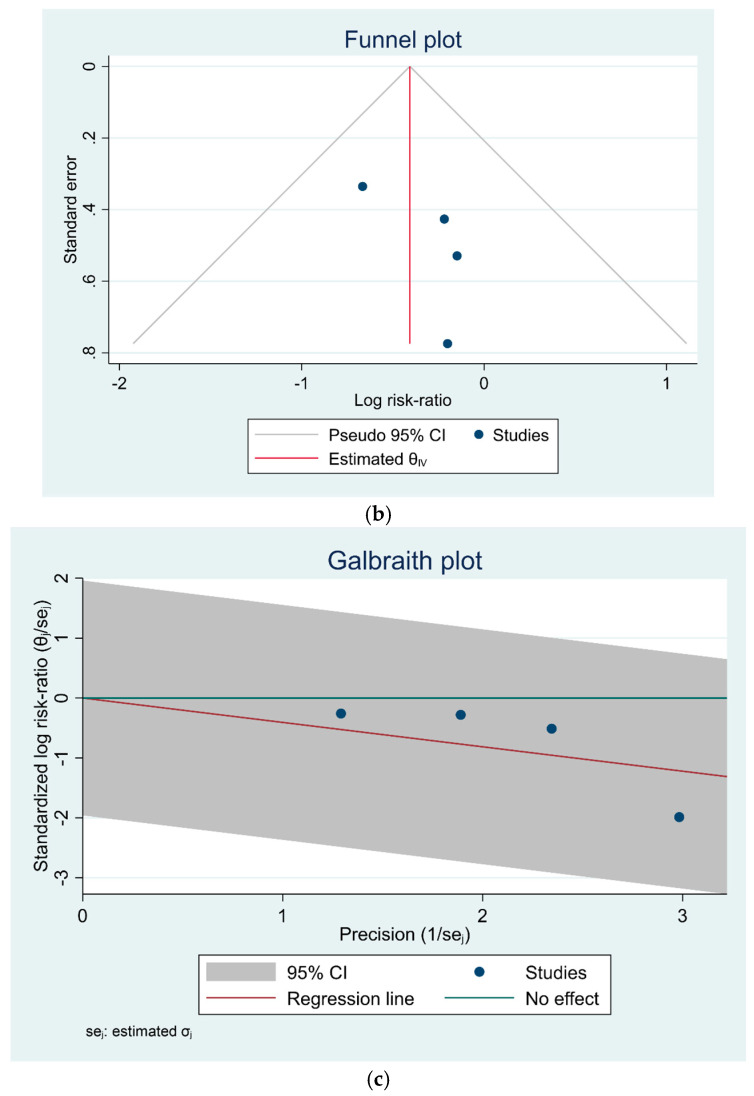
(**a**) Forest plot for gender as a risk factor of ankle sprain in soccer players. (**b**) Funnel plot for gender as a risk factor of ankle sprain in soccer players. (**c**) Galbraith plot for gender as a risk factor of ankle sprain in soccer players [28,33,35,36].

Sex did not emerge as a risk factor in any of the four articles [28,33,35,36] that explored this aspect, with relative risk figures (male to female RR = −0.41 [−0.85, 0.04]) indicating no significant difference, as shown in Figure 6a–c.

**Turf**

The role of the type of turf as a risk factor for ankle sprain was explored in six studies [23,27,39,40,46,47]. Notably, Ekstrand J. et al. [47] conducted a study between 2003 and 2005 to compare the incidence of ankle sprain on artificial turf to natural grass. In that study, cohort 1, consisting of 10 teams with artificial turf at their home facilities, showed an incidence of 4.83 ankle sprains per 1000 h of match exposure on artificial turf, compared to 2.66 on natural grass (RR = 1.81 [1–3.28]; *p* < 0.05), indicating a significant increase in risk; however, no significant difference was observed during training sessions. Conversely, in cohort 2, comprising nine teams with natural grass at their home facilities, no significant inter-cohort differences were noted, both for training (RR = 1.26 [0.58–2.73]) and matches (RR = 0.99 [0.49–2.01]). Subsequent analysis by Ekstrand J. et al. [27] extended to 2008 confirmed a significant difference in the incidence of ankle sprain between artificial turf and natural grass in men’s soccer (RR = 1.6 [1.02–2.49]; *p* < 0.05), but no discernible difference in women’s soccer was found. While Bjørneboe J. et al. [40] observed a trend towards an increased risk of ankle sprain on artificial turf during matches, this was not statistically significant. Kristenson et al., Aoki H. et al., and Soligard T. et al. [23,39,46] found no significant differences in the incidence of ankle sprains between artificial and natural turf.

**Previous ankle sprain**

**Figure 7:**
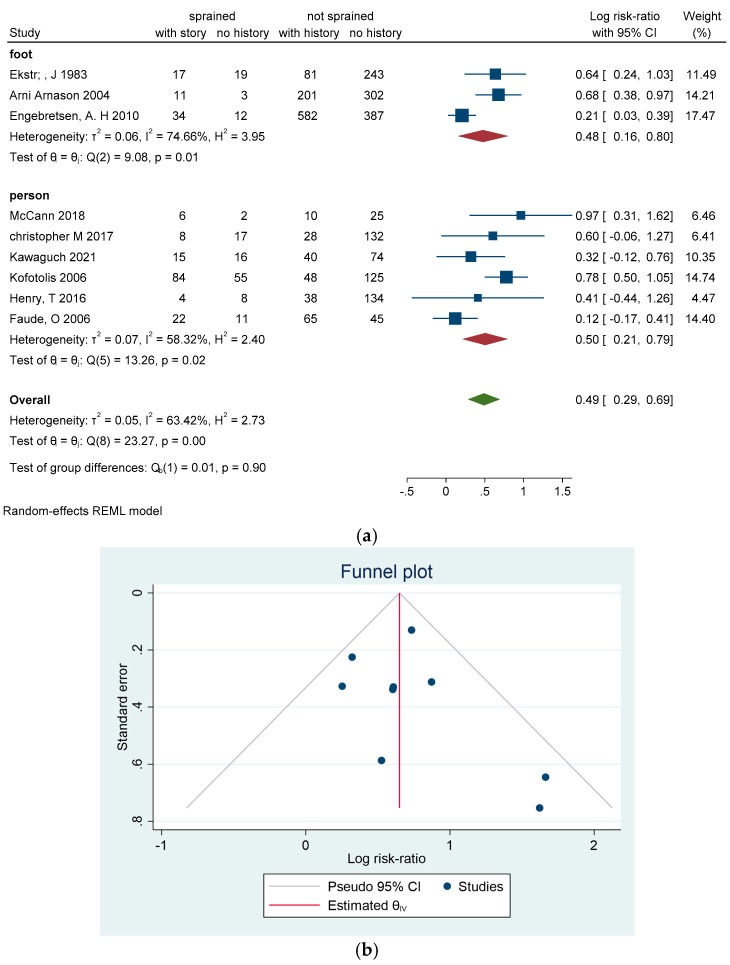

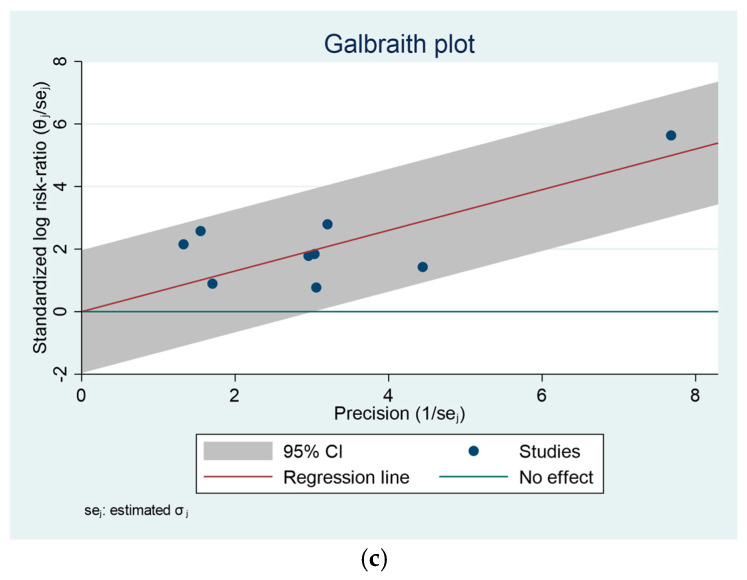
(**a**) Forest plot for previous ankle sprain as a risk factor of ankle sprain in soccer players. (**b**) Funnel plot for previous ankle sprain as a risk factor of ankle sprain in soccer players. (**c**) Galbraith plot for previous ankle sprain as a risk factor of ankle sprain in soccer players [4,18,24,26,29,30,33,34,37,38].

Previous ASI was a focus of nine studies [4,18,24,26,29,30,33,34,37,38], with five articles [4,24,26,29,37] concluding that a history of ASI significantly increases the risk of future ASI, highlighted by Hagglund M. et al. with a hazard ratio of 2.8 (CI = 0.8–9.6; *p* = 0.099), as presented in Figure 7a–c.

**Other Risk Factors**

In addition to the risk factors previously mentioned, our study revealed several other factors being evaluated for their impact on ankle sprains among soccer players (details are discussed in Table 2).

**Match congestion:** Carling C. et al. [42] explored match congestion as a potential risk factor, revealing a higher incidence of ankle sprains in the final match of two- and three-match congestion cycles compared to matches outside these congested periods.

**Hip strength and muscle force:** De Ridder et al. [25] focused on hip strength as an intrinsic risk factor for lateral ankle sprains, finding that stronger posterior chain hip muscles significantly reduced the risk. Conversely, Kawaguchi et al. [34] noted a significant difference in hip abductor muscle forces between injured and uninjured players, indicating the protective role of hip muscle strength against ankle sprains. However, they found no significant differences in knee extension, knee flexion force, muscle flexibility, or the height of the navicular tubercle between groups.

**Eccentric isokinetic strength asymmetries and GRF:** Fousekis K. et al. [31] identified eccentric isokinetic strength asymmetries in ankle dorsal and plantar flexors as a significant predictor of ankle sprains. Similarly, Fransz D. et al. [43] found that ground reaction force (GRF) in specific directions could significantly predict ankle sprains, highlighting the importance of biomechanical factors in injury risk.

**Balance tests:** Engebretsen et al. [45] did not find significant differences in balance test scores between injured and uninjured groups. However, Henry T. et al. [18] observed that poorer lower limb relative balance scores increased the risk of non-contact ankle injuries among amateur soccer players. Jupil Ko et al. [35] reported significant differences in Star Excursion Balance Test (SEBT) and Single-Leg Hop Test (SLHT) scores between injured and uninjured groups, suggesting that balance performance could influence ankle sprain risk. Kawaguchi et al. [34] found no difference between the injured and uninjured groups based on their balance measurement method.

**Dominant leg and soccer skill level:** Faude O. et al. [30] and Kofotolis et al. [4] investigated the role of limb dominance, finding that dominant legs were more prone to ankle sprains. Moreover, skill level was examined as a risk factor, with Ekstrand J. et al. [44] showing that players in higher divisions faced a greater risk of ankle injury. However, Engebretsen et al. [45] and Henry T. et al. [18] found no significant difference in ankle sprain incidence based on soccer skill level or playing experience. Longer soccer experience (years) was also found to be a risk factor for ankle sprain in a study by De Ridder et al. [25].

**Intrinsic factors:** Engebretsen A. et al. [29] and Kawaguchi et al. [34] looked into various intrinsic factors such as foot type, standing rearfoot alignment, hallux position, anterior drawer, range of motion, and ankle dorsiflexion range of motion. None of these factors showed a significant difference in the incidence of ankle sprains, indicating the complexity of accurately predicting ankle sprain risk based on intrinsic anatomical and physiological characteristics. Henry T. et al. [18] showed that poorer lower limb relative power output on vertical jump (W/Kg) was an independent risk factor for ankle sprain.

**Playing Position:** The impact of playing position on ankle sprain incidence was examined, revealing mixed results. Engebretsen et al. [29] found no significant difference in sprain rates across positions, suggesting a uniform risk. In contrast, Kofotolis et al. [4] reported that defenders had a higher injury rate than forwards and midfielders, indicating position-specific risks.

Each risk factor’s details and results are outlined in Table 2, while Table A1 assesses the included studies’ quality and Table A2 summarizes the meta-analysis outcomes according to the GRADE criteria.

## 4. Discussion

Our meta-analysis identified a history of ankle sprain as the most significant risk factor for future ankle sprain injuries (ASIs) and also has the most significant clinician implication, with body mass index (BMI) also emerging as an important contributing factor. These findings highlight the need for clinicians to closely evaluate soccer players with a history of ankle sprains or elevated BMI, as they may be at increased risk of future ASIs. Furthermore, given the prominent role of prior ankle sprains as a risk factor, further research is warranted to optimize the timing, methods, and effectiveness of rehabilitation strategies.

**Previous ankle sprain**

The recurrence of ankle sprains was identified as a substantial risk factor, with studies [24,29,37] underscoring its impact. However, Hägglund et al. [32] and Henry et al. [18] did not observe previous ankle injuries as significant, suggesting that factors such as youthfulness of the sample, competitive level, or playing conditions might play a role. Contrarily, Brinkman et al. systematic review [48] highlighted a prior ankle sprain’s role in increasing injury risk due to scar tissue formation. Moreover, it can also result in a decreased range of motion or weakened strength, indirectly impacting the likelihood of future injuries [49]. Given these findings, implementing evidence-based rehabilitation approaches is crucial to prevent recurrence and mitigate the long-term effects of prior injuries. This represents a key clinical implication of our systematic review and meta-analysis. Recent studies [50] emphasized the importance of phased rehabilitation strategies, including balance training, neuromuscular exercises, and sport-specific drills tailored to the recovery timeline. Implementing these approaches in clinical practice could significantly reduce the risk of recurrent ankle sprains and improve patient outcomes.

**Turf**

Turf quality was considered a risk factor in two studies [27,47], but Kristenson et al. [46] and Bjørneboe et al. [40] did not find a significant difference; they claimed that a lack of increase in the rate of ankle sprain could be interpreted as a continuous improvement in the quality of artificial turf playing surfaces used in football. On the other hand, Williams et al.’s [51] analysis revealed that there was evidence suggesting an elevated risk of ankle injuries when playing on artificial turf in 8 out of 14 cohorts, with incidence rate ratios ranging from 0.71 to 5.20. However, it is important to note that none of the likelihood categories reached values exceeding 95% (indicating very likely harm). Notably, they found evidence of a harmful effect associated with ankle injuries incurred during soccer matches and training on artificial turf, specifically among elite male players [27,40,47], young female players, [52], and collegiate male players during matches [53]. Conversely, a beneficial effect was inferred for soccer matches involving youths [39] and collegiate females (unlike the trivial effect during training) [53]. Artificial turf, from a biomechanical standpoint, has higher frictional coefficients compared to natural grass, leading to increased rates of foot and ankle injuries [54].

**External ankle support**

The utilization of external ankle supports plays a crucial role in preventing ankle sprains. It reduces ankle mobility, thereby potentially decreasing injury risk, but it does not impair performance in sprint speed, agility run tasks, or kicking accuracy [55,56,57,58,59,60,61,62,63]. Its efficacy is evident across different athletic groups, including male and female soccer players [64,65,66,67], professional female basketball players [68], and ballet dancers [69], especially among those with a history of ankle sprains. However, the findings from Bailey et al. [70] and Briem et al. [71] highlight that kinesiology tape may not provide the same protective benefits to healthy soccer players, pointing to the importance of selecting the appropriate type of ankle support based on the athlete’s specific needs and injury history. However, methodological differences and the utilization of different devices make direct comparisons difficult. Our interpretation is that external ankle devices can avoid the occurrence and reoccurrence of ankle sprain by providing mechanical support and increasing proprioception at the ankle and can enhance muscle response of the fibularis longus by maintaining greater levels of muscle activation, leading to a decrease in the risk of ankle sprains [62,63,67,72,73,74]. Recent reviews emphasize that the effectiveness of external ankle supports is linked to mechanical stabilization, proprioceptive enhancement, and improved activation of the fibularis longus muscle, which provides lateral stability to the ankle. Activating this muscle helps counteract inversion forces and prevent sprains, especially in individuals with chronic ankle instability [75,76,77].

**Anthropometric measurements**

Even though anthropometric characteristics did not show conclusive results, some studies performed on athletes found a relationship between BMI and the history of ankle sprain. Research conducted on football players investigating the risk factors associated with ankle sprains revealed that a high BMI and a history of previous ankle sprains, when occurring together, can significantly increase the likelihood of experiencing an ankle sprain. McHugh et al. [36] identified a history of a previous ankle sprain and a high BMI in male athletes as the only risk factors. In support of the current findings, a study conducted by Tyler et al. [78], which also investigated the correlation between past injuries and high BMI as potential risk factors among football players, indicated that players with a history of ankle sprains who were also overweight had a significantly higher injury rate, specifically 19 times greater compared to players with no prior ankle sprain and those with normal weight. Importantly, the impact of a previous ankle sprain on injury occurrence was more substantial than the effect of high BMI alone. Specifically, the injury incidence was 6.6 times higher in players with a history of ankle sprains and 3.9 times higher in those classified as overweight individuals. In a meta-analysis performed by Mason et al. [79], previous ankle sprain injury (odds ratio = 2.74, *p* < 0.001), higher body mass index (SMD = 0.50, *p* < 0.001), and higher weight (SMD = 0.24, *p* = 0.02) were identified as risk factors in male athletes. However, body characteristics did not show a significant effect on female athlete injuries. The reason that anthropometric measurements were not recognized as a risk factor was the small difference between the body mass index of the players studied in the included articles.

**Age**

We did not find age to be a strong risk factor for ankle sprain. In contrast to our findings, Faude et al. [17], discovered that younger athletes suffered from more fractures, fewer strains and sprains [80,81]. However, similar to our results, Willems et al. [82] and Powers et al. [38] did not find a significant difference. The lack of significance in our findings could be due to the sample size and the particular group that was studied.

**Limitations**

Our study has several limitations, but the main limitation is that the data reporting varied between the articles that were included. For example, for some studies, injuries were reported per hour of playing time and some injuries were reported from the number of the matches that were played. Also, in the previous ankle sprain category, some studies reported the number of feet that were injured and other studies reported the injuries by the number of players. Another limitation that can be noted is the high heterogeneity of some of the studies.

## 5. Conclusions

This study enhances our knowledge of preventing ankle sprains in soccer players. The research indicates that factors like turf quality and a history of ankle sprains are key considerations for experiencing ankle sprains. However, due to significant variations in study methodologies, additional research is necessary to identify the most effective strategies for reducing ankle sprain occurrences and relative risks in this demographic of soccer players. This study serves as a valuable resource for physicians and sports experts, aiding them in decision-making concerning warm-up protocols, preventive measures, and the assessment of playing surface quality for effective ankle injury prevention.

## Figures and Tables

**Table 1:** Risk factors of ankle sprain extracted from each paper.

	First Author Name	Year	Country	Study Design	Final Population Entered in Analysis	Male/Female	Age	Risk Factors with Details	Results
1	Aoki, H [23]	2010	Japan	Prospective Cohort	301	Not mentioned	14.5 ± 1.7 (range: 12–17)	Turf Type (Artificial Turf or Natural Grass): The inclusion criteria for players were an age between 12 and 17 years and that they had played on artificial turf (AT) for more than 1 year prior to participation in this study.	All players from the natural turf (NT) group and 52 players from the AT group (a total of 264 players) trained on NT, and all players from the AT group and 87 from the NT group (a total of 176 players) trained on AT. Of 264 players, 153 were injured while training on NT, totaling 256 acute injuries. The total number of players injured while training on AT was 66 of 176 players, totaling 169 acute injuries.
2	Arni Arnason [24]	2004	Norway	Prospective Cohort	259	All male	Mean age: 24; range: 16 to 38 years	History of Previous Ankle Sprain: The players answered a questionnaire about previous and recurrent injuries (type, location, and severity) just before the start of the season. The risk of new ankle sprains among players who previously had sustained such an injury and players with no previous injury was compared. Each leg was treated as a separate case.	11 out of 212 legs with a history and 3 out of 305 legs without a history of previous ankle sprain sustained a new ankle sprain.
3	De Ridder, R [25]	2016	Belgium	Prospective Cohort	133	All male	12.7 ± 2.1	Age and BMI: All participants played in the national league of their age category, ranging from 10 to 16 years.	A total of 133 soccer players were included for analysis. In total, 12 participants sustained a lateral ankle sprain. The *p*-values for age and BMI for sustaining ankle sprain were significant. (both < 0.001)
4	Ekstrand, J [26]	1983	Sweden	Prospective Cohort	180	All male	24.6 ± 4.6 (range: 17–38)	History of Previous Ankle Sprain: The 180 players (age = 24.6 ± 4.6, range: 17–38 years) were examined before the season for past injuries.	Of the 36 sprained ankles, 17 had been previously sprained—out of 324 ankles that did not sustain a sprain during the year, 81 had been previously sprained. Thus, ankle sprains were significantly more common (*p* < 0.01) in those with a previous sprain.
5	Ekstrand, J [27]	2011	Sweden	Prospective Cohort	767	613 male/154 female	25 ± 5 (range: 16–38)	Turf Type (Artificial Turf or Natural Grass): Twenty-five elite teams (nineteen male and six female teams) that had reported the installation of a third-generation artificial turf pitch to UEFA were invited to participate. In total, 767 (613 male and 154 female) players were included.	There were no significant differences in the overall incidence of injury between the surfaces during training or match play for either males or females. There were also no significant differences between artificial turf and grass when the incidences were compared in terms of injury severity sub-categories.
6	Emery, C [28]	2005	Canada	Prospective Cohort	317	153 male/164 female	14.89 for males/14.75 for females (range: 12–18)	Gender: Competitive soccer players in Alberta are divided into age groups of 2 years each (under 18, 16, and 14) and by skill level (divisions 1–4), in which division 1 is the most elite division of play. One team for each gender, division (1–4), and age group from the Calgary Blizzard Soccer Club was randomly selected to participate in this study.	12 ankle sprain (rate = 1.73) occurred in girls and 9 (rate = 1.28) in boys.
7	Engebretsen, A. H [29]	2010	Norway	Prospective Cohort	508	All male	24.0 (range: 16.2–37.7)	History of Previous Ankle Sprain, Height, Weight, Age, and BMI: 508 players were tested for potential risk factors for ankle injuries during the 2004 preseason, January through March, at the Norwegian School of Sport Sciences. Every player capable (not injured at the time) completed single-leg balance tests for both legs (both on a balance mat and on the floor), a clinical examination, and a questionnaire.	Univariate analyses revealed the number of previous acute ankle injuries as a potential leg-dependent risk factor for acute ankle injuries. None of the balance tests (floor or balance mat) or clinical tests were candidates for predicting an increased risk of ankle injury. Additionally, none of the player-dependent factors (age, height, body mass index, position on the field, having played at the junior national team level or at the senior national team level, level of play this season, or level of play the previous season) were significantly associated with the risk of ankle injury.
8	Faude, O [30]	2006	Germany	Prospective Cohort	143	All female	22.4 (5.0) years	History of Previous Ankle Sprain: 143 players (22.4 (5.0) years of age, 61 (60) kilograms, and 169 (6) centimeters—values shown as mean (SD)) provided baseline information as well as complete data on injuries and exposure times. They were followed over a whole outdoor season from August 2003 to mid-June 2004, including preseason conditioning. Baseline information was recorded at the start of the season for each player by the physiotherapist.	Players with previous sprains of the ankle had a slightly, but not significantly, higher risk of the same injury. When each leg was treated as a separate case, no higher risk of an actual sprain in players with a previous sprain was found. The OR (95%CI) was 0.71 (0.31 to 1.62; *p* = 0.42).
9	Fousekis, K [31]	2012	Greece	Prospective Cohort	100	Not mentioned	23.6 (4.2)	Age, BMI, Weight, and Generalized Joint Hypermobility: A cohort of 100 players was recruited from 4 third division professional soccer teams. The players were screened for inclusion in this study if they sustained no injury for at least a period of 6 months before testing. A preseason evaluation of the ankle joint was conducted for isokinetic muscle strength, flexibility, joint stability, neuromuscular coordination, and anthropometric characteristics.	Seventeen (70.8%) of the ligament injuries in the ankle joint were non-contact lateral sprains; the logistic regression analysis revealed 3 significant predictors of non-contact ankle sprains: (A) eccentric isokinetic strength asymmetries of ankle dorsal and plantar flexors (OR = 8.88; 95% CI, 1.95–40.36; *p* = 0.005), (B) increased BMI (OR = 8.16; 95% CI, 1.42–46.63; *p* = 0.018), and (C) increased body weight (OR = 5.72; 95% CI, 1.37–23.95; *p* = 0.017).
10	Hägglund, M [32]	2006	Sweden	Prospective Cohort	197	All male	25 ± 4 years (range: 17–38)	History of Previous Ankle Sprain, Height, Weight, and Age: 197 players who participated in both seasons were included (mean (SD) values: age: 25 (4) years (range: 17–38), height: 182 (5) centimeters (range: 170–197), and weight: 79 (6) kilograms (range: 65–98)). The baseline variables used in the risk factor analysis in season 2002 were (a) prospectively recorded injuries in season 2001 and (b) anthropometrics (age, height, weight, and body mass index (BMI))	Previous injury, age, height, and weight were all associated with ankle sprain in the univariate analysis (BMI *was not found to be a risk factor for ankle sprain*, *p* > 0.2). In the multivariate model, there was a tendency towards an increase in risk for ankle sprain in the previously injured leg and a decrease in risk for ankle sprain with increasing age, but none of the variables reached statistical significance.
11	Henry, T [18]	2016	Australia	Prospective Cohort	210	All male	18.9 ± 3.5	History of Previous Ankle Sprain, Height, Weight, Age, and BMI: Participants were excluded if they were younger than 15 years, showed signs or symptoms of illness, or had an injury preventing them from completing preseason screening. Before preseason testing, each of the participants completed a questionnaire to identify their age, injury history, and team competition level.	None of them showed a significant effect on ankle sprain.
12	Emery, C. A [33]	2010	Canada	RCT	380 intervention and 364 control	Intervention: 161 female/219 male Control: 251 female/113 male	13–18 years	History of Previous Ankle Sprain, Age, and Gender: The main outcome measure was a warm-up intervention program, but these 3 factors were also measured as risk factors for ankle sprain.	History of previous ankle sprain: adjusted incidence rate ratio (95% CI) (previous sprain vs. no previous sprain) = 2.29 (1.23 to 4.28) *p* < 0.05. Age: (U15–U18) vs. (U13–U15): adjusted incidence rate ratio (95% CI) = 2.62 (1.14 to 6.0) *p* < 0.05 Gender: gender was not a significant risk factor for any injury definition; however, point estimates suggest a greater risk for females for ankle sprain (IRR = 1.86 (95% CI: 0.72 to 4)).
13	Kawaguchi [34]	2021	Japan	Prospective Cohort	145	All male	Injured: 19.9 ± 1.0 Uninjured: 19.8 ± 1.2	BMI, Age, and Isometric Hip Abduction: The results indicated that inversion ankle sprain was significantly associated with hip abductor strength.	In this season, there were 31 inversion ankle sprains (21.4%) in 31 players. Only isometric hip abductor strength was considerably lower in injured players compared to unaffected ones. A logistic regression analysis identified hip abductor muscle strength deficiency as a significant risk factor for inversion ankle sprain (odds ratio, 0.978 [95% CI, 0.976–0.999]; *p*= 0.05).
14	Jupil Ko [35]	2018	USA	Prospective Cohort	64	Injured: 5 males and 7 females; uninjured: 24 male and 28 female	Injured: 16.1 ± 1.4 Uninjured: 15.4 ± 1.3	Age, Gender, Height, BMI, and Mass: There were no significant differences in age, height, mass, and BMI between the injured and the uninjured groups.	A total of 64 participants (age = 15.5 ± 1.3 years; height = 161.7 ± 7.7 cm; and mass = 57.1 ± 8.4 kg) were recruited from a junior soccer club and monitored for 10 months.
15	Kofotolis [4]	2006	Greece	Prospective Cohort	312	All male	24.8 ± 4.63	History, Position, and Exposure Time: Multinomial logistic regression showed that previous ankle sprain (*p* < 0.05) is a significant predictor of ankle sprain injury.	The injury rate was higher in the first two months of the season compared to the last month (*p* < 0.05). Using multinomial logistic regression, previous ankle sprain was found to be a significant predictor of injury (*p* < 0.05).
16	McHugh [36]	2006	USA	Prospective Cohort	60	33 male and 27 female	16 ± 1	Balance Test, Hip Abduction Strength, Hip Adduction Strength, and History of Previous Ankle Sprain: At the beginning of each season, all athletes completed preseason physical examinations that included measurements of height; weight; BMI; strength in hip flexion, abduction, and adduction; balance in single-limb stance; and generalized ligamentous laxity.	The incidence of grade II and grade III sprains was higher in athletes with a history of a previous ankle sprain.
17	McCann [37]	2018	Australia	Prospective Cohort	43	All female	19.7 ± 1.1	History of Previous Ankle Sprain, BMI, Mass, and Height: Participants who sustained an LAS (n = 8) were significantly taller than those who did not sustain an LAS (n = 35). A logistic regression analysis (odds ratio = 1.30 [1.00, 1.70]) and area under the ROC curve analysis (AUROC = 0.73 [0.58, 0.89], *p* = 0.04) further exhibited the predictive value of height. A logistic regression analysis (odds ratio = 1.87 [1.22, 1.98]) exhibited the predictive value of previous ankle sprain history. Mass and BMI demonstrated no predictive value for LAS.	Taller collegiate women’s soccer players and those with previous ankle sprain history may have a greater predisposition to LAS.
18	Christopher M [38]	2017		Prospective Cohort	185	All male	Injured: 20.9 ± 5.9 Uninjured: 19.6 ± 5.1	Age, Height, Mass, BMI, Previous History, and Hip Abductor Strength: Baseline hip abductor strength was lower in injured players than in uninjured players (*p*: 0.008). Logistic regression indicated that impaired hip abductor strength increased the future injury risk (OR: 1.10 [95% CI: 1.02–1.18], *p*: 0.010).	Reduced isometric hip abductor strength predisposed competitive male soccer players to non-contact lateral ankle sprains.
19	Soligard T [39]	2012	Norway	Prospective Cohort	Not mentioned	Not mentioned	13–19	Turf Type: While there was no difference in the risk of ankle sprains between the two surfaces (rate ratio: 0.39; 95% CI: 0.12–1.23), the risk of ankle injuries overall was almost half on artificial turf compared to grass.	
20	Bjørneboe J [40]	2010	Norway	Prospective Cohort	Not mentioned	All male	Not mentioned	Turf Type: 48 ankle sprains on grass and 17 on artificial turf (artificial versus grass IRR = 0.83 (0.48–1.44).	A trend towards an increased risk of knee and ankle sprains on artificial turf was observed, albeit only during matches.
21	Vieira [41]	2012		Prospective Cohort	83	All male	14–19	Joint Hypermobility: A total of 43 cases of ankle injury due to sprains were recorded, of which 9 episodes were in players with JHS, thus making *p* = 0.106. The significance level was 5%.	There was insufficient evidence to assert that there is an association with an increased incidence of ankle sprains among patients with JHS.
22	Emery C [28]	2005	Canada	Prospective Cohort	317	153 male/164 female	14.89 for males and 14.75 for females	Sex: Ankle sprain injury rate was 1.73 in girls and 1.28 in boys.	
23	Carling, C [42]	2015	United Kingdom	Prospective Cohort	Not mentioned	All male	Not mentioned	Match Congestion: Risk of ankle sprain risk during 2 consecutive matches separated by a short time interval of ≤72 h, 3 consecutive matches during 96 h, and matches outside these congestion cycles.	There was a higher risk of ankle sprain in the final match in the two-match congestion cycles (IRR = 5.4 [1.0–29.3]; *p* = 0.0522) and three-match congestion cycles (IRR = 10.4 [1.9–57.9]; *p* = 0.0068) compared to matches played outside these congested cycles.
24	Fransz D [43]	2018	Netherlands	Prospective Cohort	190 (cohort 1 from 2012 to 2015 = 138; cohort 2 from 2013 to 2016 = 52)	All male	U13 (n = 34): 11.8 ± 0.6 U15 (n = 45): 13.9 ± 0.6 U17 (n = 43): 15.7 ± 0.8 U19 (n = 44): 17.7 ± 0.7 First and second (n = 24): 23.2 ± 3.2	Ground Reaction Force (GRF): They measured GRF in the vertical, anteroposterior, and mediolateral directions in a single-legged drop-jump landing from 20 cm height in 190 male soccer players and followed them to measure the incidence of ankle sprain.	The root mean square of the GRF in the mediolateral direction with regard to the first 0.4 s after landing (RMS ML: 0.4) was found to be a significant predictor of ankle sprain (*p* = 0.017). Horizontal GRF during the late dynamic phase (3.0–5.0 s) (Hor GRF late dyn) had a significant predictive capacity for ankle sprain as well (*p* = 0.029). In the multivariate analysis with regard to the prediction of all ankle sprains, the RMS ML: 0.4 and Hor GRF late dyn were combined into a significant risk factor model (*p* = 0.005).
25	Ekstrand. J [44]	1990	Sweden	Prospective Cohort	639	All male	25 ± 4 (range: 17–38)	Soccer Skill Level: They followed 41 soccer teams from 4 different skill levels (with division 1 being the highest-skill group and division 6 being the lowest-skill group).	There was a significant difference in the incidence of ankle sprains/team between divisions 2 and 4 (*p* < 0.05), but the *p*-value of the difference between other divisions was not significant. Players in the higher divisions are at higher risk for ankle injury during a season because of longer exposure time. The higher injury rate during matches for high-level players is probably due to intensity, speed, etc., which differs between divisions. The higher injury rate during practice for low-level players may be due to factors such as bad training conditions, as well as physical differences among the players.
26	Engebretsen, A [45]	2008	Norway	Prospective Cohort	508	All male	Not mentioned	Balance: players were asked to stand barefoot on one straight leg and maintain this position only using their ankle joint to correct balance. Soccer Skill Level: they studied the effect of the level of soccer play on the incidence of ankle sprain.	No significant difference between the injured and uninjured group was detected regarding balance test scores (*p* = 0.64 for balance score on the floor and *p* = 0.41 for balance score on the mat). No significant differences between the 1st and 2nd divisions (2nd to 1st OR = 1.08 [0.5–2.34]; *p* = 0.85) and between 1st and 3rd divisions (3rd to 1st OR = 0.89 [0.36–2.21]; *p* = 0.8) were found.

AT: Artificial Turf, NT: Natural Turf, LAS: Lateral Ankle Sprain, JHS: Joint Hypermobility Syndrome, GRF: Ground Reaction Force, RMS ML: Root Mean Square in the Mediolateral Direction, Hor GRF late dyn: Horizontal GRF during the Late Dynamic Phase, BMI: Body Mass Index, SD: Standard Deviation, IRR: Incidence Rate Ratio, OR: Odds Ratio, CI: Confidence Interval, and ROC: Receiver Operating Characteristic Curves.

**Table 2:** Details of the other risk factors of ankle sprain in soccer players.

	Author	Risk Factors	Details	Results	Conclusion
1	Carling, C [42]	Match Congestion	Risk of ankle sprain during 2 consecutive matches separated by a short time interval of ≤72 h, 3 consecutive matches during 96 h, and matches outside these congestion cycles.	There was a higher risk of ankle sprain in the final match in the two-match congestion cycles (IRR = 5.4 [1.0–29.3]; *p* = 0.0522) and three-match congestion cycles (IRR = 10.4 [1.9–57.9]; *p* = 0.0068) compared to matches played outside these congested cycles.	Match congestion is a risk factor for ankle sprain incidence.
2	De Ridder [25]	Hip Muscle Force	They measured the strength of the flexors, extensors, abductors, adductors, and internal and external rotators of the hip.	They only identified posterior chain hip muscle force as an independent risk factor for a lateral ankle sprain (HR = 0.3 [0.1–0.9]; *p* = 0.028).	Players with stronger posterior chain hip muscles had significantly lower hazards of sustaining a lateral ankle sprain.
3	Kawaguchi [34]	Hip and Knee Muscle Force, Muscle Flexibility, and Height of the Navicular Tubercle	They measured knee extension, knee flexion, and hip abduction strength in 145 soccer players. They also investigated muscle flexibility (iliopsoas, quadriceps femoris, hamstrings, gastrocnemius, and soleus muscles) and the height of the navicular tubercle in each player’s foot.	The odds of a male collegiate soccer player sustaining an inversion ankle sprain were increased by approximately 2% for each 1 N.m (Newton.meter) decrease in hip abductor strength (OR = 0.978 [0.976–0.999]; *p* = 0.047).	The only significant difference in muscle forces between injured and uninjured players was for hip abductors.
4	Fousekis, K [31]	Muscle Strength, Flexibility, Joint Stability, and Neuromuscular Coordination	A preseason evaluation of the ankle joint was conducted for isokinetic muscle strength, flexibility, joint stability, neuromuscular coordination, and anthropometric characteristics.	Eccentric isokinetic strength asymmetries of ankle dorsal and plantar flexors were significant predictors of non-contact ankle sprains (OR = 8.88 [1.95–40.36]; *p* = 0.005).	Soccer players with preseasonal eccentric strength asymmetries (15%) in the ankle joint had 8.8 times the odds of sustaining a non-contact ankle sprain than did players with no eccentric strength asymmetry in the same joint at the same period. Other factors, such as muscle flexibility and proprioceptive traits, do not seem to affect ankle sprain occurrence.
5	Fransz D [43]	Ground Reaction Force (GRF)	They measured GRF in the vertical, anteroposterior, and mediolateral directions in a single-legged drop-jump landing from 20 cm height in 190 male soccer players and followed them to measure the incidence of ankle sprain.	The root mean square of the GRF in the mediolateral direction with regard to the first 0.4 s after landing (RMS ML: 0.4) was found to be a significant predictor of ankle sprain (*p* = 0.017). Horizontal GRF during the late dynamic phase (3.0–5.0 s) (Hor GRF late dyn) had a significant predictive capacity for ankle sprain as well (*p* = 0.029). In the multivariate analysis with regard to the prediction of all ankle sprains, the RMS ML of 0.4 and Hor GRF late dyn were combined into a significant risk factor model (*p* = 0.005).	The root mean square of the GRF in the mediolateral direction during the first 0.4 s after landing (RMS ML: 0.4) and the mean resultant horizontal GRF during the late dynamic phase (3.0–5.0 s; Hor GRF late dyn) following a single-legged drop-jump landing are related to the occurrence of a lateral ankle sprain among male elite soccer players.
6	Engebretsen [45]	Balance	999 players were asked to stand barefoot on one straight leg and maintain this position only using their ankle joint to correct balance.	No significant difference between the injured and uninjured group was detected regarding balance test scores (*p* = 0.64 for balance score on the floor and *p* = 0.41 for balance score on the mat).	Balance tests do not increase our ability to identify players at risk.
7	Henry T. [18]	Balance	They used an electronic board to measure the balance score while standing with both legs on the board in 67 soccer players.	The results showed a significant difference in balance test scores (*p* = 0.015).	Poorer lower limb relative balance scores are associated with an increased risk of non-contact ankle injury among amateur soccer players.
8	Jupil Ko [35]	Balance	They measured Star Excursion Balance Test (SEBT) and Single-Leg Hop Test (SLHT) scores in a cohort of 64 soccer players.	They reported a significant difference between injured (n = 12) and uninjured (n = 52) groups (*p* < 0.05).	Adolescent soccer players who sustained a lateral ankle sprain(s) demonstrated shorter SEBT-posteromedial and SEBT-posterolateral reach distances and a longer completion time in the SLHT.
9	Kawaguchi [34]	Balance	They measured double- and single-leg stances with a 1 m Footscan pressure plate, and the total distance of the center of pressure during the 30 s of standing on the plate in both tests was measured as the balance parameter.	They found no difference between the injured and uninjured groups (42 ± 27.1 vs. 41.6 ± 20.9, respectively; *p* = 0.53)	
10	Faude O. [30]	Leg Dominance	143 female soccer players sustained 41 ankle sprains in 10 months.	27 ankle sprains occurred in dominant legs and 14 in non-dominant legs (kai2 = 4.122, *p* = 0.04).	They found a significant difference in ankle sprains according to limb dominance.
11	Kofotolis [4]	Leg Dominance		The dominant legs sustained 68.3% of all ankle injuries (*p* < 0.05).	They found a significant difference in ankle sprains according to limb dominance.
12	Ekstrand J. [44]	Soccer Skill Level	They followed 41 soccer teams from 4 different skill levels (with division 1 being the highest-skill group and division 6 being the lowest-skill group).	There was a significant difference in the incidence of ankle sprains/team between divisions 2 and 4 (*p* < 0.05), but the *p*-value of the difference between other divisions was not significant.	Players in the higher divisions are at higher risk for ankle injury during a season because of longer exposure time. The higher injury rate during matches for high-level players is probably due to intensity, speed, etc., which differs between divisions. The higher injury rate during practice for low-level players may be due to factors such as bad training conditions, as well as physical differences among the players.
13	Engebretsen [45]	Soccer Skill Level	They studied the effect of the level of soccer play on the incidence of ankle sprain.	No significant differences between the 1st and 2nd divisions (2nd to 1st OR = 1.08 [0.5–2.34]; *p* = 0.85) and between 1st and 3rd divisions (3rd to 1st OR = 0.89 [0.36–2.21]; *p* = 0.8) were found.	
14	Henry T. [18]	Soccer Skill Level	They studied the effect of the level of soccer play on the incidence of ankle sprain.	High competition level to low-level ankle sprain RR = 1.81 [0.65–5.04]; *p* = 0.247	They found a non-significant difference between different skill levels.
15	De Ridder [25]	Soccer Experience (Years)	They studied the effect of years of soccer experience on the incidence of ankle sprain.	Ankle sprain group experience was 9.1 ± 1.8 years, and the rest of the players’ experience was 7.2 ± 1.9 years (*p* = 0.004).	Longer soccer experience (years) was found to be a risk factor for ankle sprain.
16	Engebretsen A. [29]	Foot Anatomy and Dynamics	They studied foot type (normal, pes planus, pes cavus, and splayed forefoot), standing rearfoot alignment (normal and valgus), hallux position (normal, valgus), anterior drawer (normal, pathologic), and range of motion for supination, pronation, and dorsiflexion as intrinsic risk factors for ankle sprain.		None of them showed a significant difference in the incidence of ankle sprain.
17	Henry T. [18]	Foot Anatomy and Dynamics		There was a higher incidence of ankle sprain in soccer players with an ankle dorsiflexion range of motion of more than 13 cm, but the *p*-value was not great enough to prove that it was a risk factor (RR = 3.49 [0.73–16.6]; *p* = 0.142). However, they could prove poorer lower limb relative power output on vertical jump (W/Kg) as an independent risk factor for ankle sprain (RR = 6.24 [0.82–47.32]; *p* = 0.038).	
18	Kawaguchi [34]	Foot Anatomy and Dynamics	They investigated ankle dorsiflexion range of motion (degrees) as a risk factor for ankle sprain.	There was not a significant difference between injured and uninjured limbs in injured players (40.4 ± 7.1 vs. 40.4 ± 5.7; *p* = 0.85), and there was no difference between injured and uninjured players in the ankle dorsiflexion range of motion (40.4 ± 7.1 vs. 39.6 ± 6.3; *p* = 0.50).	
19	Engebretsen [29]	Playing Position	They compared the risk of ankle sprain in different positions with forward players.	Attacking midfielders had the greatest odds of sustaining an ankle injury (OR = 1.93 [0.63–5.87]; *p* = 0.25) and goalkeepers had the lowest odds (OR = 0.3 [0.03–2.53]; *p* = 0.27), but the difference between positions was not significant.	
20	Kofotolis [4]	Playing Position		Goalkeepers had the lowest injury rate, and defenders had a greater injury rate compared to forwards and midfielders. Only the *p*-value for the higher rate of injury in defenders was significant (<0.05).	

GRF: Ground Reaction Force, RMS ML: Root Mean Square in the Mediolateral Direction, Hor GRF late dyn: Horizontal GRF during the Late Dynamic Phase, SEBT: Star Excursion Balance Test, SLHT: Single-Leg Hop Test, BMI: Body Mass Index, SD: Standard Deviation, IRR: Incidence Rate Ratio, HR: Hazard Ratio, OR: Odds Ratio, CI: Confidence Interval, and RR: Risk Ratio.

## Data Availability

Available based upon reasonable request to the corresponding author.

## Appendix A

**Search Protocol**

**Databases**

1. MEDLINE/PubMed
2. Scopus
3. CENTRAL
4. Web of Science
5. ProQuest

**Restrictions**

No language, filter, or date restriction

Search strategy

**#1** Ankle [Mesh]

**#2** Ankle Joint [Mesh]

**#3** Lateral Ligament, Ankle [Mesh]

**#4** Syndesmos*

**#5** External Lateral Ligament

**#6** #1 OR #2 OR #3 OR #4 OR #5

**#7** Sprains and Strains [Mesh]

**#8** sprain*

**#9** strain*

**#10** rupture*

**#11** Instabilit*

**#12** unstable

**#13** function*

**#14** #7 OR #8 OR #9 OR #10 OR #11 OR #12 OR #13

**#15** #6 AND #14

**#16** Ankle Injuries [Mesh]

**#17** #15 OR #16

**#18** Risk Factors [Mesh]

**#19** Risk Evaluation and Mitigation [Mesh]

**#20** Risk*

#21 Relative risk

#22 Incidence*

#23 Epidemiolog*

#24 Survey

#25 Patterns

#26 Prevalence*

**#27** #18 OR #19 OR #20 OR #21 OR #22 OR #23 OR #24 OR #25 OR #26

**#28** Soccer [Mesh]

**#29** Football [Mesh]

**#30** Athlet*

**#31** Sport*

**#32** #28 OR #29 OR #30 OR #31

**#33** #17 AND #27 AND #32

**Specific issues:**

[Mesh] only can be used in PubMed, Central.

Central

[((([mh Ankle] OR [mh “Ankle Joint”] OR [mh “Lateral Ligament, Ankle”] OR “Ankle Syndesmos*” OR “External Lateral Ligament”) AND ([mh Sprains and Strains] OR sprain* OR strain* OR rupture OR Instabilit* OR unstable OR function*)) OR ([mh“Ankle Injuries”])) AND ([mh“Risk Factors”] OR [mh Risk Evaluation and Mitigation] OR Risk* OR Relative risk OR Incidence* OR Epidemiolog* OR Survey OR Pattern* OR Prevalence*) AND ([mh Football] OR [mh soccer] OR Sport* OR Athlet*)]:ti,ab,kw

**MEDLINE (through PUBMED)**

(((Ankle [mh] OR “Ankle Joint” [mh] OR “Lateral Ligament, Ankle” [mh] OR “Ankle Syndesmos*” OR “External Lateral Ligament”) AND (Sprains and Strains [Mesh] OR sprain* OR strain* OR rupture OR Instabilit* OR unstable OR function*)) OR (“Ankle Injuries” [mh])) AND (“Risk Factors” [mh] OR Risk Evaluation and Mitigation [Mesh] OR Risk* OR Relative risk OR Incidence* OR Epidemiolog* OR Survey OR Pattern* OR Prevalence*) AND (Football [Mesh] OR Soccer [Mesh] OR Sport* OR Athlet*)

**Web of Science**

(((TS=(((Ankle OR “Ankle Joint” OR “Lateral Ligament, Ankle” OR “Ankle Syndesmos*” OR “External Lateral Ligament”) AND (Sprains and Strains OR sprain* OR strain* OR rupture OR Instabilit* OR unstable OR function*)) OR (“Ankle Injuries”)) AND (“Risk Factors” OR Risk Evaluation and Mitigation OR Risk* OR Relative risk OR Incidence* OR Epidemiolog* OR Survey OR Pattern* OR Prevalence*) AND (Football OR Soccer OR Sport* OR Athlet*)))

**Scopus**

(((Ankle OR “Ankle Joint” OR “Lateral Ligament, Ankle” OR “Ankle Syndesmos”* OR “External Lateral Ligament”) AND (Sprains and Strains [Mesh] OR sprain* OR strain* OR rupture OR Instabilit* OR unstable OR function*)) OR (“Ankle Injuries”)) AND (“Risk Factors” OR Risk Evaluation and Mitigation OR Risk* OR Relative risk OR Incidence* OR Epidemiolog* OR Survey OR Pattern* OR Prevalence*) AND (Football OR Soccer OR Sport* OR Athlet*)

## Appendix B

**Table A1:** Quality assessment of cohort studies based on the JBI tool.

Author	Year	Study Design	Q1	Q2	Q3	Q4	Q5	Q6	Q7	Q8	Q9	Q10	Q11
Aoki, H [23]	2010	prospective cohort	yes	yes	yes	unclear	yes	yes	yes	yes (1 year)	unclear	unclear	yes
Arni Arnason [24]	2004	prospective cohort	yes	yes	yes	yes	yes	yes	yes	yes (4 months)	yes	yes	yes
Carling, C [42]	2015	prospective cohort	yes	yes	yes	no	no	yes	yes	yes (6 seasons)	unclear	no	yes
De Ridder, R [25]	2016	prospective cohort	yes	yes	yes	yes	yes	yes	yes	yes (3 seasons)	yes	yes	yes
Soligard T [39]	2012	prospective cohort	yes	yes	unclear	unclear	not applicable	yes	unclear	yes (3 seasons)	yes	unclear	yes
Ekstr, J [26]	1983	prospective cohort	unclear	yes	yes	no	no	yes	yes	yes (12 months)	unclear	no	yes
Ekstr, J [27]	2011	prospective cohort	yes	yes	yes	yes	yes	yes	yes	yes (February 2003 to October 2008)	no	yes	yes
Ekstr, J [47]	2006	prospective cohort	yes	yes	yes	yes	yes	yes	yes	yes (2003–2004 season)	no	yes	yes
Ekstr, J [47]	1990	prospective cohort	yes	yes	yes	no	no	yes	yes	yes (12 months)	unclear	no	yes
Emery, C [28]	2005	prospective cohort	yes	yes	yes	unclear	no	yes	yes	yes (13 weeks)	yes	unclear	yes
Engebretsen, A. H [29]	2010	prospective cohort	yes	yes	yes	yes	yes	yes	yes	yes	yes	yes	yes
Faude, O [30]	2006	prospective cohort	yes	yes	yes	yes	unclear	yes	yes	yes (10 months)	yes	yes	yes
Fousekis, K [31]	2012	prospective cohort	yes	yes	yes	yes	yes	yes	yes	yes (10 months)	yes	yes	yes
Fransz, D [43]	2018	prospective cohort	yes	yes	yes	yes	yes	yes	yes	yes	yes	yes	yes
Hägglund, M [32]	2006	prospective cohort	yes	yes	yes	yes	yes	yes	yes	yes (2 seasons: 2001–2002)	yes	yes	yes
Henry, T [18]	2016	prospective cohort	yes	yes	yes	yes	yes	yes	yes	yes (2 seasons: 2008–2009)	unclear	no	yes
Kristenson [46]	2013	prospective cohort	no	yes	unclear	unclear	not applicable	yes	yes	2010–2011	yes	no	yes
Bjørneboe J [40]	2010	prospective cohort	no	yes	yes	unclear	not applicable	yes	yes	yes 2004–2007	yes	unclear	yes
McHugh [36]	2006	prospective cohort	yes	yes	yes	yes	yes	yes	yes	2 years	yes	not applicable	yes
McCann [37]	2018	prospective cohort	yes	yes	yes	yes	unclear	yes	yes	unclear	yes	not applicable	yes
Christopher M [38]	2017	prospective cohort	yes	yes	yes	yes	unclear	yes	yes	2 years	yes	not applicable	yes
Kawaguchi [34]	2021	prospective cohort	yes	yes	yes	yes	unclear	yes	yes	2019 season	yes	not applicable	yes
Kofotolis [4]	2006	prospective cohort	yes	yes	yes	yes	unclear	yes	yes	2 years	yes	not applicable	yes
Vieira [41]	2012	prospective cohort	yes	yes	unclear	yes	unclear	yes	unclear	2009 season	yes	not applicable	unclear
Jupil ko [35]	2018	prospective pilot study	yes	yes	yes	yes	unclear	yes	yes	2014/2015	yes	not applicable	yes

**Table A2:** Quality assessment of RCT studies based on Cochrane tools.

Author	Random Sequence Generation	Allocation Concealment	Blinding of Participants and Personnel	Blinding of Outcome Assessment	Incomplete Outcome Data	Selective Reporting	Other Bias
Emery C 2010 [33]	yes	yes	yes	yes	No	probably	not detected

## References

[1] Junge A., Dvorak J., Graf-Baumann T. (2004). Football injuries during the World Cup 2002. Am. J. Sports Med..

[2] Cloke D.J., Spencer S., Hodson A., Deehan D. (2009). The epidemiology of ankle injuries occurring in English Football Association academies. Br. J. Sports Med..

[3] Hootman J.M., Dick R., Agel J. (2007). Epidemiology of collegiate injuries for 15 sports: Summary and recommendations for injury prevention initiatives. J. Athl. Train..

[4] Kofotolis N.D., Kellis E., Vlachopoulos S.P. (2007). Ankle sprain injuries and risk factors in amateur soccer players during a 2-year period. Am. J. Sports Med..

[5] Fong D.T.-P., Hong Y., Chan L.-K., Yung P.S.-H., Chan K.-M. (2007). A systematic review on ankle injury and ankle sprain in sports. Sports Med..

[6] Andersen T.E., Floerenes T.W., Arnason A., Bahr R. (2004). Video analysis of the mechanisms for ankle injuries in football. Am. J. Sports Med..

[7] Palmer-Green D.S., Batt M.E., Scammell B.E. (2016). Simple advice for a simple ankle sprain? The not so benign ankle injury. Osteoarthr. Cartil..

[8] Verhagen E., Van Der Beek A., Twisk J., Bouter L., Bahr R., Van Mechelen W. (2004). The effect of a proprioceptive balance board training program for the prevention of ankle sprains: A prospective controlled trial. Am. J. Sports Med..

[9] Emery C., Roos E.M., Verhagen E., Finch C., Bennell K., Story B., Spindler K., Kemp J., Lohmander L. (2015). OARSI clinical trials recommendations: Design and conduct of clinical trials for primary prevention of osteoarthritis by joint injury prevention in sport and recreation. Osteoarthr. Cartil..

[10] Valderrabano V., Hintermann B., Horisberger M., Fung T.S. (2006). Ligamentous posttraumatic ankle osteoarthritis. Am. J. Sports Med..

[11] Whittaker J.L., Woodhouse L., Nettel-Aguirre A., Emery C. (2015). Outcomes associated with early post-traumatic osteoarthritis and other negative health consequences 3–10 years following knee joint injury in youth sport. Osteoarthr. Cartil..

[12] Emery C.A., Roy T.-O., Whittaker J.L., Nettel-Aguirre A., Van Mechelen W. (2015). Neuromuscular training injury prevention strategies in youth sport: A systematic review and meta-analysis. Br. J. Sports Med..

[13] Hägglund M., Atroshi I., Wagner P., Waldén M. (2013). Superior compliance with a neuromuscular training programme is associated with fewer ACL injuries and fewer acute knee injuries in female adolescent football players: Secondary analysis of an RCT. Br. J. Sports Med..

[14] Olsen O.-E., Myklebust G., Engebretsen L., Holme I., Bahr R. (2005). Exercises to prevent lower limb injuries in youth sports: Cluster randomised controlled trial. BMJ.

[15] Owoeye O.B., Akinbo S.R., Tella B.A., Olawale O.A. (2014). Efficacy of the FIFA 11+ warm-up programme in male youth football: A cluster randomised controlled trial. J. Sports Sci. Med..

[16] Soligard T., Nilstad A., Steffen K., Myklebust G., Holme I., Dvorak J., Bahr R., Andersen T.E. (2010). Compliance with a comprehensive warm-up programme to prevent injuries in youth football. Br. J. Sports Med..

[17] Faude O., Rößler R., Junge A. (2013). Football injuries in children and adolescent players: Are there clues for prevention?. Sports Med..

[18] Henry T., Evans K., Snodgrass S.J., Miller A., Callister R. (2016). Risk factors for noncontact ankle injuries in amateur male soccer players: A prospective cohort study. Clin. J. Sport. Med..

[19] Liberati A., Altman D.G., Tetzlaff J., Mulrow C., Gøtzsche P.C., Ioannidis J.P., Clarke M., Devereaux P.J., Kleijnen J., Moher D. (2009). The PRISMA statement for reporting systematic reviews and meta-analyses of studies that evaluate health care interventions: Explanation and elaboration. Ann. Intern. Med..

[20] Ouzzani M., Hammady H., Fedorowicz Z., Elmagarmid A. (2016). Rayyan—A web and mobile app for systematic reviews. Syst. Rev..

[21] Porritt K., Gomersall J., Lockwood C. (2014). JBI’s systematic reviews: Study selection and critical appraisal. AJN Am. J. Nurs..

[22] Wan X., Wang W., Liu J., Tong T. (2014). Estimating the sample mean and standard deviation from the sample size, median, range and/or interquartile range. BMC Med. Res. Methodol..

[23] Aoki H., Kohno T., Fujiya H., Kato H., Yatabe K., Morikawa T., Seki J. (2010). Incidence of injury among adolescent soccer players: A comparative study of artificial and natural grass turfs. Clin. J. Sport Med..

[24] Arnason A., Sigurdsson S.B., Gudmundsson A., Holme I., Engebretsen L., Bahr R. (2004). Risk factors for injuries in football. Am. J. Sports Med..

[25] De Ridder R., Witvrouw E., Dolphens M., Roosen P., Van Ginckel A. (2017). Hip strength as an intrinsic risk factor for lateral ankle sprains in youth soccer players: A 3-season prospective study. Am. J. Sports Med..

[26] Ekstrand J., Gillquist J. (1983). Soccer injuries and their mechanisms: A prospective study. Med. Sci. Sports Exerc..

[27] Ekstrand J., Hägglund M., Fuller C. (2011). Comparison of injuries sustained on artificial turf and grass by male and female elite football players. Scand. J. Med. Sci. Sports.

[28] Emery C.A., Meeuwisse W.H., Hartmann S.E. (2005). Evaluation of risk factors for injury in adolescent soccer: Implementation and validation of an injury surveillance system. Am. J. Sports Med..

[29] Engebretsen A.H., Myklebust G., Holme I., Engebretsen L., Bahr R. (2010). Intrinsic risk factors for acute ankle injuries among male soccer players: A prospective cohort study. Scand. J. Med. Sci. Sports.

[30] Faude O., Junge A., Kindermann W., Dvorak J. (2006). Risk factors for injuries in elite female soccer players. Br. J. Sports Med..

[31] Fousekis K., Tsepis E., Vagenas G. (2012). Intrinsic risk factors of noncontact ankle sprains in soccer: A prospective study on 100 professional players. Am. J. Sports Med..

[32] Hägglund M., Waldén M., Ekstrand J. (2006). Previous injury as a risk factor for injury in elite football: A prospective study over two consecutive seasons. Br. J. Sports Med..

[33] Emery C., Meeuwisse W. (2010). The effectiveness of a neuromuscular prevention strategy to reduce injuries in youth soccer: A cluster-randomised controlled trial. Br. J. Sports Med..

[34] Kawaguchi K., Taketomi S., Mizutani Y., Inui H., Yamagami R., Kono K., Takagi K., Kage T., Sameshima S., Tanaka S. (2021). Hip abductor muscle strength deficit as a risk factor for inversion ankle sprain in male college soccer players: A prospective cohort study. Orthop. J. Sports Med..

[35] Ko J., Rosen A.B., Brown C.N. (2018). Functional performance tests identify lateral ankle sprain risk: A prospective pilot study in adolescent soccer players. Scand. J. Med. Sci. Sports.

[36] McHugh M.P., Tyler T.F., Tetro D.T., Mullaney M.J., Nicholas S.J. (2006). Risk factors for noncontact ankle sprains in high school athletes: The role of hip strength and balance ability. Am. J. Sports Med..

[37] McCann R.S., Kosik K.B., Terada M., Beard M.Q., Buskirk G.E., Gribble P.A. (2018). Acute lateral ankle sprain prediction in collegiate women’s soccer players. Int. J. Sports Phys. Ther..

[38] Powers C.M., Ghoddosi N., Straub R.K., Khayambashi K. (2017). Hip strength as a predictor of ankle sprains in male soccer players: A prospective study. J. Athl. Train..

[39] Soligard T., Bahr R., Andersen T.E. (2012). Injury risk on artificial turf and grass in youth tournament football. Scand. J. Med. Sci. Sports.

[40] Bjørneboe J., Bahr R., Andersen T.E. (2010). Risk of injury on third-generation artificial turf in Norwegian professional football. Br. J. Sports Med..

[41] Vieira R.B., Bertolini F.M., Vieira T.C., Aguiar R.M., Pinheiro G.B., Lasmar R.C.P. (2012). Incidence of ankle sprains in soccer players with joint hypermobility syndrome. Rev. Bras. Ortop..

[42] Carling C., McCall A., Le Gall F., Dupont G. (2016). The impact of short periods of match congestion on injury risk and patterns in an elite football club. Br. J. Sports Med..

[43] Fransz D.P., Huurnink A., Kingma I., de Boode V.A., Heyligers I.C., van Dieën J.H. (2018). Performance on a single-legged drop-jump landing test is related to increased risk of lateral ankle sprains among male elite soccer players: A 3-year prospective cohort study. Am. J. Sports Med..

[44] Ekstrand J., Tropp H. (1990). The incidence of ankle sprains in soccer. Foot Ankle.

[45] Engebretsen A.H., Myklebust G., Holme I., Engebretsen L., Bahr R. (2008). Prevention of injuries among male soccer players: A prospective, randomized intervention study targeting players with previous injuries or reduced function. Am. J. Sports Med..

[46] Kristenson K., Bjørneboe J., Waldén M., Andersen T.E., Ekstrand J., Hägglund M. (2013). The Nordic Football Injury Audit: Higher injury rates for professional football clubs with third-generation artificial turf at their home venue. Br. J. Sports Med..

[47] Ekstrand J., Timpka T., Hägglund M. (2006). Risk of injury in elite football played on artificial turf versus natural grass: A prospective two-cohort study. Br. J. Sports Med..

[48] Brinkman R.E., Evans T.A. (2011). History of ankle sprain as a risk factor of future lateral ankle sprain in athletes. J. Sport. Rehabil..

[49] Garrett W.E. (1996). Muscle strain injuries. Am. J. Sports Med..

[50] Tedeschi R., Ricci V., Tarantino D., Tarallo L., Catani F., Donati D. (2024). Rebuilding Stability: Exploring the Best Rehabilitation Methods for Chronic Ankle Instability. Sports.

[51] Williams S., Hume P.A., Kara S. (2011). A review of football injuries on third and fourth generation artificial turfs compared with natural turf. Sports Med..

[52] Steffen K., Andersen T.E., Bahr R. (2007). Risk of injury on artificial turf and natural grass in young female football players. Br. J. Sports Med..

[53] Fuller C.W., Dick R.W., Corlette J., Schmalz R. (2007). Comparison of the incidence, nature and cause of injuries sustained on grass and new generation artificial turf by male and female football players. Part 1: Match injuries. Br. J. Sports Med..

[54] Griffith G.G., Brett A.G., Gregory P.G., Heath P.G., Thomas Robert W., Stanislaw P.S. (2022). Playing Surface and Injury Risk: Artificial Turf vs. Natural Grass. Chapter 3: Injuries and Sports Medicine.

[55] Bocchinfuso C., Sitler M.R., Kimura I.F. (1994). Effects of Two Semirigid Prophylactic Ankle Stabilizers on Speed, Agility, and Vertical Jump. J. Sport Rehabil..

[56] Greene T.A., Wight C.R. (1990). A comparative support evaluation of three ankle orthoses before, during, and after exercise. J. Orthop. Sports Phys. Ther..

[57] Gross M.T., Everts J.R., Roberson S.E., Roskin D.S., Young K.D. (1994). Effect of Donjoy Ankle Ligament Protector and Aircast Sport-Stirrup orthoses on functional performance. J. Orthop. Sports Phys. Ther..

[58] Macpherson K., Sitler M., Kimura I., Horodyski M. (1995). Effects of a semirigid and softshell prophylactic ankle stabilizer on selected performance tests among high school football players. J. Orthop. Sports Phys. Ther..

[59] Paris D.L., Sullivan S.J. (1992). Isometric strength of rearfoot inversion and eversion in nonsupported, taped, and braced ankles assessed by a hand-held dynamometer. J. Orthop. Sports Phys. Ther..

[60] Gross M.T., Liu H.Y. (2003). The role of ankle bracing for prevention of ankle sprain injuries. J. Orthop. Sports Phys. Ther..

[61] Putnam A.R., Bandolin S.N., Krabak B.J. (2012). Impact of Ankle Bracing on Skill Performance in Recreational Soccer Players. PM&R.

[62] Burks R.T., Bean B.G., Marcus R., Barker H.B. (1991). Analysis of athletic performance with prophylactic ankle devices. Am. J. Sports Med..

[63] Bot S.D., van Mechelen W. (1999). The effect of ankle bracing on athletic performance. Sports Med..

[64] Tropp H., Askling C., Gillquist J. (1985). Prevention of ankle sprains. Am. J. Sports Med..

[65] Surve I., Schwellnus M.P., Noakes T., Lombard C. (1994). A fivefold reduction in the incidence of recurrent ankle sprains in soccer players using the Sport-Stirrup orthosis. Am. J. Sports Med..

[66] Ekstrand J., Gillquist J., Liljedahl S.-O. (1983). Prevention of soccer injuries: Supervision by doctor and physiotherapist. Am. J. Sports Med..

[67] Sharpe S.R., Knapik J., Jones B. (1997). Ankle braces effectively reduce recurrence of ankle sprains in female soccer players. J. Athl. Train..

[68] Kofotolis N., Kellis E. (2007). Ankle sprain injuries: A 2-year prospective cohort study in female Greek professional basketball players. J. Athl. Train..

[69] Botsis A.E., Schwarz N.A., Harper M.E., Liu W., Rooney C.A., Gurchiek L.R., Kovaleski J.E. (2019). Effect of Kinesio(^®^) Taping on Ankle Complex Motion and Stiffness and Jump Landing Time to Stabilization in Female Ballet Dancers. J. Funct. Morphol. Kinesiol..

[70] Bailey D., Firth P. (2017). Does kinesiology taping of the ankles affect proprioceptive control in professional football (soccer) players?. Phys. Ther. Sport.

[71] Briem K., Eythörsdöttir H., Magnúsdóttir R.G., Pálmarsson R., Rúnarsdöttir T., Sveinsson T. (2011). Effects of kinesio tape compared with nonelastic sports tape and the untaped ankle during a sudden inversion perturbation in male athletes. J. Orthop. Sports Phys. Ther..

[72] Callaghan M.J. (1997). Role of ankle taping and bracing in the athlete. Br. J. Sports Med..

[73] Fong D.T., Chan Y.Y., Mok K.M., Yung P.S., Chan K.M. (2009). Understanding acute ankle ligamentous sprain injury in sports. Sports Med. Arthrosc. Rehabil. Ther. Technol..

[74] Olmsted L.C., Vela L.I., Denegar C.R., Hertel J. (2004). Prophylactic Ankle Taping and Bracing: A Numbers-Needed-to-Treat and Cost-Benefit Analysis. J. Athl. Train..

[75] Dizon J.M., Reyes J.J. (2010). A systematic review on the effectiveness of external ankle supports in the prevention of inversion ankle sprains among elite and recreational players. J. Sci. Med. Sport.

[76] Feger M.A., Donovan L., Hart J.M., Hertel J. (2015). Lower extremity muscle activation in patients with or without chronic ankle instability during walking. J. Athl. Train..

[77] Mendez-Rebolledo G., Guzmán-Venegas R., Cruz-Montecinos C., Watanabe K., Calatayud J., Martinez-Valdes E. (2024). Individuals with chronic ankle instability show altered regional activation of the peroneus longus muscle during ankle eversion. Scand. J. Med. Sci. Sports.

[78] Tyler T.F., Mchugh M.P., Mirabella M.R., Mullaney M.J., Nicholas S.J. (2006). Risk factors for noncontact ankle sprains in high school football players: The role of previous ankle sprains and body mass index. Am. J. Sports Med..

[79] Mason J., Kniewasser C., Hollander K., Zech A. (2022). Intrinsic Risk Factors for Ankle Sprain Differ Between Male and Female Athletes: A Systematic Review and Meta-Analysis. Sports Med. Open.

[80] Bahr R., Krosshaug T. (2005). Understanding injury mechanisms: A key component of preventing injuries in sport. Br. J. Sports Med..

[81] van Mechelen W., Hlobil H., Kemper H.C. (1992). Incidence, severity, aetiology and prevention of sports injuries. A review of concepts. Sports Med..

[82] Willems T.M., Witvrouw E., Delbaere K., Philippaerts R., De Bourdeaudhuij I., De Clercq D. (2005). Intrinsic risk factors for inversion ankle sprains in females—A prospective study. Scand J. Med. Sci. Sports.

